# Cerebellar Transcranial Direct Current Stimulation in Children with Developmental Coordination Disorder: A Randomized, Double-Blind, Sham-Controlled Pilot Study

**DOI:** 10.1007/s10803-021-05202-6

**Published:** 2021-07-27

**Authors:** Haifa Akremi, Raphaël Hamel, Anne Dumas, Chantal Camden, Hélène Corriveau, Jean-Francois Lepage

**Affiliations:** 1 School of Rehabilitation, Sherbrooke University, 3001-12th Ave. North, Sherbrooke, QC Canada; 2 Department of Pediatrics, Sherbrooke University, 3001-12th Ave. North, Sherbrooke, QC Canada; 3 Sherbrooke University Research Center, 3001-12th Ave. North, Sherbrooke, QC Canada; 4 Department of Pediatrics, Faculty of Medicine and Health Sciences, Sherbrooke University, 3001-12th Avenue North, Sherbrooke, QC J1H 5N4 Canada

**Keywords:** Neurostimulation, Neurodevelopmental disorders, Motor learning, Cerebellum, Transcranial direct current stimulation (tDCS)

## Abstract

Evidence-based therapeutic options for children with developmental coordination disorder (DCD) are scarce. This work explored the effects of cerebellar anodal transcranial direct current stimulation (atDCS) on three 48 h-apart motor sequence learning and upper limb coordination sessions in children with DCD. The results revealed that, as compared to a Sham intervention (n = 10), cerebellar atDCS (n = 10) did not meaningfully improve execution speed but tended to reduce the number of execution errors during motor sequence learning. However, cerebellar atDCS did neither meaningfully influence offline learning nor upper limb coordination, suggesting that atDCS’ effects are circumscribed to its application duration. These results suggest that cerebellar atDCS could have beneficial effects as a complementary therapeutic tool for children with DCD.

## Introduction

Developmental coordination disorder (DCD) is a prevalent neurodevelopmental condition affecting 5–6% of school-age children characterized by motor learning and coordination difficulties independent of other medical or intellectual disorders (American Psychiatric Association, 2013). These motor impairments significantly impact daily activities and are associated with debilitating physical and mental health impacts (O’Dea & Connell, 2016). Motor learning difficulties appear to be central to the challenges experienced by children with DCD. In fact, DCD is often presented as a motor learning disorder (Biotteau et al., 2016; Schoemaker & Smits-engelsman, 2015), as children show delays in achieving developmental milestones, such as learning to cycle, handwrite or tie shoelaces, and require more time, repetition, and feedback than their peers to perform motor tasks.

There are currently few evidence-based therapeutic options for DCD (Smits-Engelsman et al., 2018). The most common forms of intervention are task-oriented approaches, in which children practice real-world tasks, learn to elaborate motor plans, and identify and correct their errors. These approaches combining practice with cognitive strategies are widely used, but a recent meta-analysis of 15 studies offers mitigated support for this type of intervention, as the only two randomized controlled trials conducted so far failed to show the benefits of task-based interventions on motor performance (Miyahara et al., 2020). This lack of therapeutic options calls for the development of new methods to alleviate the impairments of children with DCD.

To date, the neurological basis of motor learning impairments in children with DCD remains poorly understood. Recent studies using functional magnetic resonance imagery (fMRI) in DCD have shown abnormal recruitment of several brain regions typically involved in motor learning, planning, and performance, including the dorsolateral prefrontal cortex, the precentral gyrus, and parietal cortex, all of which are part of the cortico-cerebellar loop (Kelly & Strick, 2003; Zwicker et al., 2011). Namely, a consensual body of literature underlines the importance of the cerebellum in motor learning and coordination in healthy adults (Caligiore et al., 2019; Celnik, 2015; De Zeeuw & Ten Brinke, 2015), but also the motor learning and coordination impairments in DCD children (Zwicker et al., 2012). Namely, recent neuroimaging studies have found functional alterations in the crus I, lobule VI, and lobule IX of the cerebellum’s posterior lobe of DCD children (Debrabant et al., 2013; Zwicker et al., 2011), indicating that the cerebellum is a prime target for neurorehabilitation of children with DCD (Brown-Lum & Zwicker, 2015).

Transcranial direct current stimulation (tDCS) is a non-invasive brain stimulation technique that relies on the application of a weak electric current (2 mA) to modulate brain activity (Celnik, 2015). Painless and safe, tDCS is widely used and is considered to bear minimal risks in the school-aged population (Bikson et al., 2016). Typical tDCS relies on a two-electrode montage; a current travels from the anode to the cathode, resulting in increased excitability in the region located under the anode (Nitsche & Paulus, 2000). In healthy individuals, this so-called anodal tDCS (atDCS) applied to the cerebellum has been shown to enhance motor learning and retention (Buch et al., 2017; Cantarero et al., 2015; Giordano et al., 2017; Jalali et al., 2018; O’Brien et al., 2018; Shimizu et al., 2017; Wessel et al., 2016; but see Ballard et al. (2019)). The positive impact of cerebellar atDCS on motor skills has also been shown in clinical conditions such as cerebellar ataxia (Benussi et al., 2017; Grimaldi et al., 2016; Pozzi et al., 2014) and cerebral palsy (Grecco et al., 2017). However, it remains to be established whether this type of neuromodulatory approach may also prove beneficial for individuals with DCD.

The primary aim of the study was to assess the efficacy of cerebellar atDCS over three learning sessions in modulating motor sequence learning and retention in children with DCD. Secondary aims included the assessment of the effects of cerebellar atDCS and motor sequence learning on upper limb coordination. It was hypothesized that active cerebellar atDCS, in comparison to sham atDCS stimulation, would improve motor sequence learning by increasing learning speed and accuracy, as measured from a classic serial reaction time task (SRTT) (Robertson, 2007; Robertson et al., 2004).

## Methods

### Participants

**Fig. 1:**
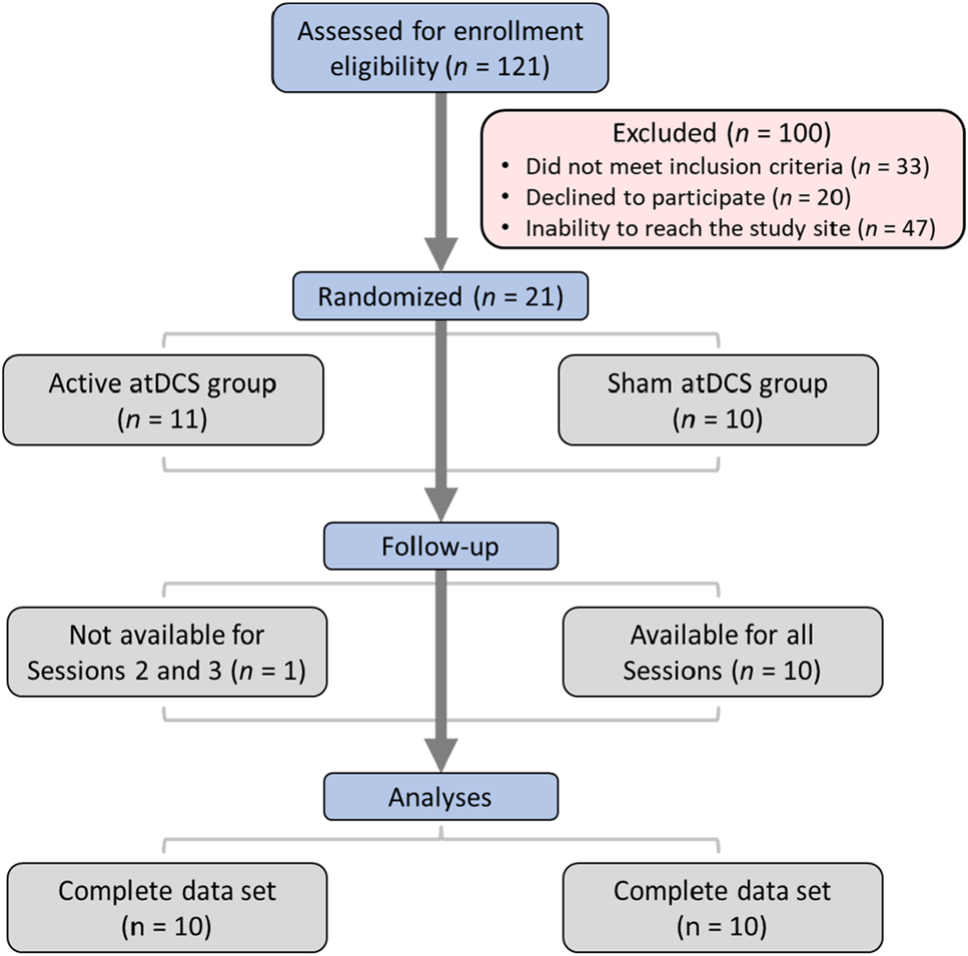
Flow diagram of participants’ enrollment. A total of 121 participants were initially assessed for eligibility, but only 20 participants with complete data sets remained for the statistical analyses

A sample of 121 children participants was initially assessed for eligibility, but only 21 of them successfully met eligibility criteria (Fig. 1). One further participant had to be rejected because his participation in all three sessions was not completed, which left a total sample size of 20 children participants (Fig. 1). All participants were recruited via public ads and medical archives of the institution. Children were eligible to participate if they were aged between 10 and 16 years old, right-handed or ambidextrous (Edinburgh Handedness Inventory score ≥ − 50; (Oldfield, 1971)), and had a confirmed medical diagnostic of DCD. Exclusion criteria included all contraindication for tDCS (Ferrucci et al., 2013) or the existence of comorbid neurodevelopmental, neurological, or psychiatric conditions, except for attention deficit hyperactivity disorder (ADHD) because of its high concomitance with DCD (Piek et al., 2007; Tal Saban et al., 2014). Written informed consent was obtained from all parents and children gave verbal assent. This study was approved by the institutional ethics board and was conducted per the 1964 Declaration of Helsinki.

### Study Design and Protocol

**Fig. 2:**
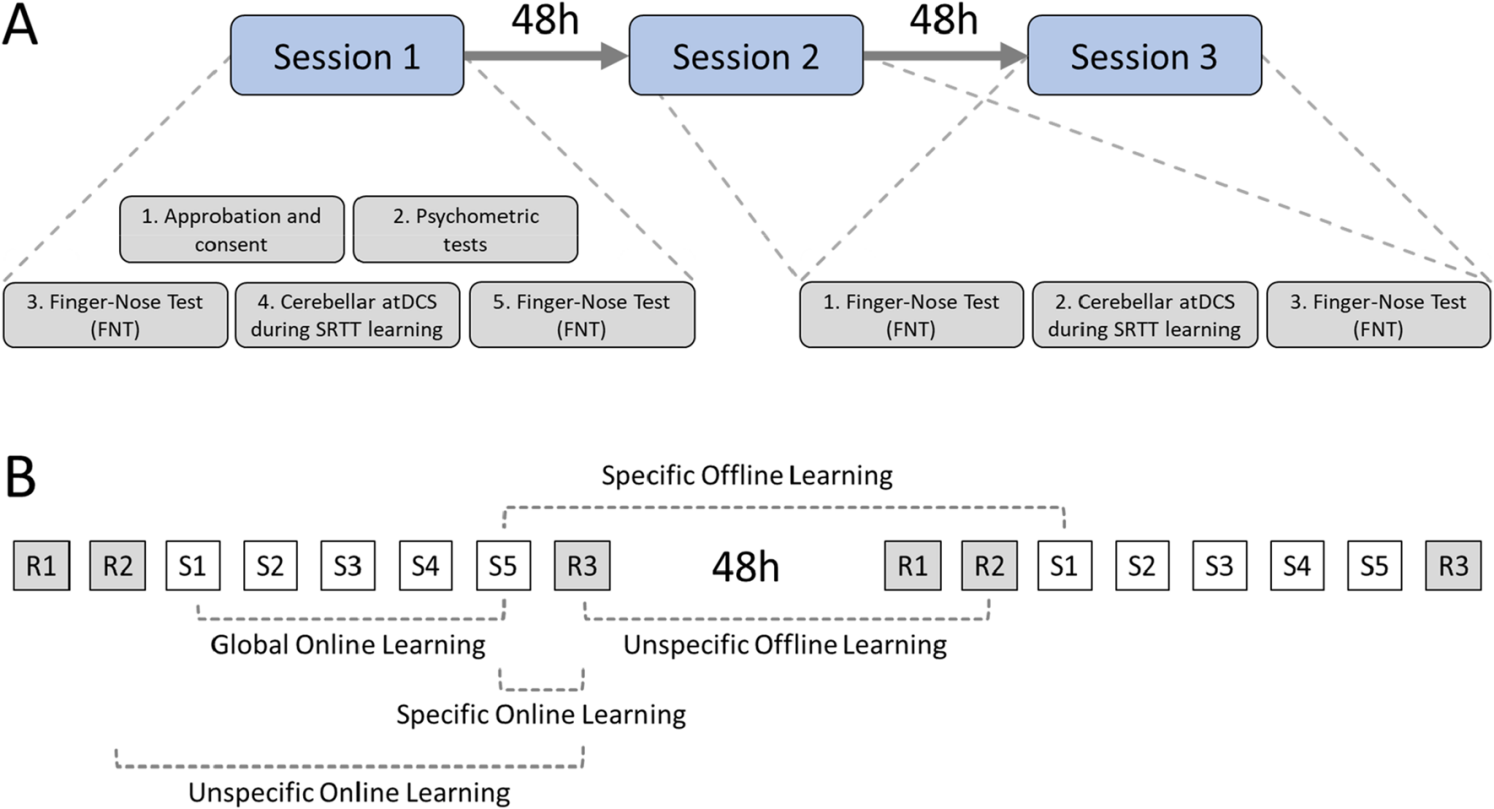
Overview of study design. **a** Procedures of each experimental session. Note that Session 2 and Session 3 were identical. **b** Overview of the different contrasts calculated to assess learning during the SRTT task. Note that only two SRTT sessions are represented. Blocks beginning with “R” or “S” denote random and sequence blocks, respectively

The study consisted of a double-blind, randomized sham-controlled experiment. After confirming their eligibility, participants were randomly assigned to the experimental (i.e. Active atDCS) or sham (i.e. Sham atDCS) group using computerized random blocks. The study protocol was the same for both groups and included three stimulation sessions that occurred at 48 h intervals (Fig. 2A). Group assignment and the application of cerebellar atDCS were performed by a research assistant that was not involved in data collection or analysis. Children, parents, and all other experimenters were kept blind until the end of data collection. Before the first session, the level of motor impairment was evaluated using Movement Assessment Battery of Children-2nd edition (MABC-2) (Henderson et al., 2007), which uses a percentile rank to categorize the severity of coordination difficulties in children. The impact of DCD on daily activities was evaluated using the French Canadian version of the Developmental Coordination Disorder Questionnaire (DCD-Q) (Fritsch et al., 2010) filled out by the parent, where the total score indicates a suspicion or not of DCD. Before and after each stimulation session, upper limb coordination was assessed using the Finger Nose Test (FNT).

### Motor Coordination: Finger Nose Test (FNT)

The FNT (Swaine et al., 2005), a clinical test with excellent reliability in children with and without DCD was used (Peters et al., 2008; Wilson et al., 1992). Eyes closed, participants are asked to touch their nose with their index finger, then fully extend the arm at shoulder level, and repeat the movement five times as fast and as accurately as possible. Participants were allowed 10 practice attempts before performing the test. Completion time and accuracy (number of times successfully touching their nose) were used as variables. The FNT was performed before and after each cerebellar atDCS session.

### Serial Reaction Time Task (SRTT)

Motor learning was assessed with the SRTT, a computerized task commonly used to evaluate motor learning in neuromodulation studies (Ciechanski & Kirton, 2016; Nissen & Bullemer, 1987). In this task, four squares are shown on a computer screen, each corresponding to a specific key on the keyboard. For each trial, a given square was blacked-out and participants were instructed to press the correct key with the corresponding finger of their right hand as fast as possible (with the index to the little fingers set to “J”, “K”, “L”, and “:” keys, respectively). Reaction time is calculated as the interval between the onset of the stimuli and the pressing of the key, and an error is registered when a non-target key is pressed. The time interval between the response and the presentation of the next stimulus was fixed at 0 ms because short intervals prevent gaining explicit knowledge of the repeated sequence (Destrebecqz et al., 2005). During each session, eight blocks consisting of 120 trials were initially performed with the right hand. The first, second, and eighth blocks consisted of stimuli presented in random order (Blocks R1, R2, R3). The third to the seventh blocks (S1, S2, S3, S4, S5) consisted of a 12-stimuli sequence (4-2-3-1-1-3-2-1-3-4-2-4) repeated ten times to induce motor learning. Subjects were allowed to take a few minutes rest between blocks to alleviate fatigue. As it is usually the case with the SRTT (Jongkees et al., 2019; Morin-Parent et al., 2017; Perez et al., 2007), the first random block (R1) was used to familiarize participants with the task, and the second random block (R2) was used as a baseline to monitor individual learning performance. Since they acted as familiarization runs, the R1 blocks of every session were discarded from further analyses. The same approach was used for reaction time and errors. Trials with reaction time under 250 ms or exceeding 2.5 standard deviations from the mean of each block were excluded from the analysis, as they are not representative of a reaction-based response (Ciechanski & Kirton, 2016). Stimuli presentation and data recordings were managed with Superlab 5 software (Cedrus, California, CA).

### Transcranial Direct Current Stimulation

tDCS was administered simultaneously with the SRTT using an HDCKit device (Magstim, UK) for 20 min during each session, following the parameters and montage described in previously published protocols (Cantarero et al., 2015; Ehsani et al., 2016; Ferrucci et al., 2013). Briefly, the current was delivered through two sponge electrodes soaked in 0.9% saline solution; a 35 cm^2^ (7 × 5 cm) anode was centered on the median line 2 cm below the inion of the occipital bone (Ferrucci et al., 2013), and a 25 cm^2^ (5 × 5 cm) cathode was positioned over the left shoulder. The electrodes were held in place with two rubber straps, one around the head and the other on the left arm. In the active atDCS condition, the current intensity was set at 2 mA with a 30-s ramp-up and ramp-down. In the sham condition, the device was turned off after the initial ramp-up. This blinding procedure was effective, as groups did not differ regarding the correct guessing of their experimental condition (Pearson’s χ^2^; χ^2^ = 0.833, *p* = 0.361).

### Statistical Analyses

Regarding the SRTT data, the RT and Error data of all sessions were normalized (%) to the random block R2 of the first session. Then, the following online (within-sessions) contrasts were calculated (Fig. 2B). Global online learning was assessed by calculating the RT and Error difference between the last (S5) and first (S1) sequence blocks of the same session. Specific online learning was assessed by calculating the RT and Error difference between the last sequence (S5) and random (S3) blocks of the same session. Unspecific online learning was assessed by calculating the RT and Error difference between the last (R3) and second (R2) random blocks of the same session. Offline (between-sessions) contrasts were also calculated. Namely, specific offline learning was assessed by calculating the RT and Error difference between the first (S1) and last (S5) sequence blocks of the subsequent and preceding sessions, respectively. Unspecific offline learning was assessed by calculating the RT and Error difference between the second (R2) and last (R3) random blocks of the subsequent and preceding sessions, respectively. The resulting contrasts were submitted to omnibus tests. Namely, mixed two-way ANOVAs with Sessions as a within-subject factor and Groups as the between-subject factor were conducted on RT and Error data. Concerning FNT data, mixed three-way ANOVAs were conducted on the Time and Number data, with Sessions and Times (Pre, Post) as within-subject and Groups as a between-subject factor.

Given the exploratory nature of this work (de Groot, 2014; Wagenmakers et al., 2012), both significant (defined as *p* < 0.05) and marginal (defined as 0.05 < *p* < 0.10 and when the effect size is above the large ${n}_{p}^{2}$ or Cohen’s d (dz) benchmark values of 0.140 and 0.800, respectively (Lakens, 2013)) main effects and interactions were decomposed using pairwise comparisons. The Shapiro–Wilk test was used to assess normality (Razali & Wah, 2011) and the Benjamini–Hochberg procedure (Benjamini & Hochberg, 1995) was used to correct for inflated type 1 errors due to multiple comparisons (Ludbrook, 1998). Deviations from normality (*p* < 0.05; Shapiro–Wilk test) resulted in the use of Wilcoxon’s and Mann–Whitney U’s rank test instead of dependent and independent t-tests, respectively. Statistical analyses were performed with *jamovi* 1.2.6 (www.jamovi.org).

## Results

### Similar Participants’ Characteristics in the Two Groups

Participants’ characteristics are shown in Table 1. Groups did not differ significantly regarding age, gender, laterality, co-occurrence of ADHD or other disorders, and the use of psychostimulant medications. The DCD-Q (*t*_(18)_ = 0.411, *p* = 0.686, Cohen’s d = 0.184) and the MABC-2 scores (*t*_(18)_ = 0.052, *p* = 0.959, Cohen’s d = 0.012) were similar across groups. The overall sample was representative of the DCD population, with 65% being males and 65% having ADHD (Piek et al., 2007; Tal Saban et al., 2014). No serious adverse effects occurred during or after the study completion.

**Table 1:** Mean and SEM, where applicable, of participant’s characteristics

	Active atDCS group (*n* = 10)	Sham atDCS group (*n* = 10)	Tests used	p values
Age (years)	12.10 ± 0.64	12.70 ± 0.60	Ind. T-test	0.502
Sex	7 males; 3 females	6 males; 4 females	Pearson’s χ^2^	0.639
Laterality (Edinburgh score/100)	88.00 ± 9.98%	96.10 ± 2.60%	Mann–Whitney U*	0.914
Diagnosis of ADHD	7 out of 10	6 out of 10	Pearson’s χ^2^	0.639
Diagnosis of other learning disorders	2 out of 10	1 out of 10	Pearson’s χ^2^	0.607
Use of psychostimulant medications	7 out of 10	6 out of 10	Pearson’s χ^2^	0.639
DCD-Q score	37.90 ± 2.83	36.10 ± 3.33	Ind. T-test	0.686
MABC-2 score	15.91 ± 6.5	15.50 ± 4.48	Ind. T-test	0.959

“ADHD” refers to Attention deficit hyperactivity disorder. “DCD-Q” refers to the Developmental Coordination Disorder Questionnaire. “MABC-2” refers to the Movement Assessment Battery of Children-2nd edition. “Ind.” refers to Independent. The asterisk refers to a deviation of normality (Shapiro–Wilk; p < 0.05). The p values have not been corrected for multiple comparisons

### RT Data: No Reliable atDCS-Induced Improvements During SRTT Learning

**Fig. 3:**
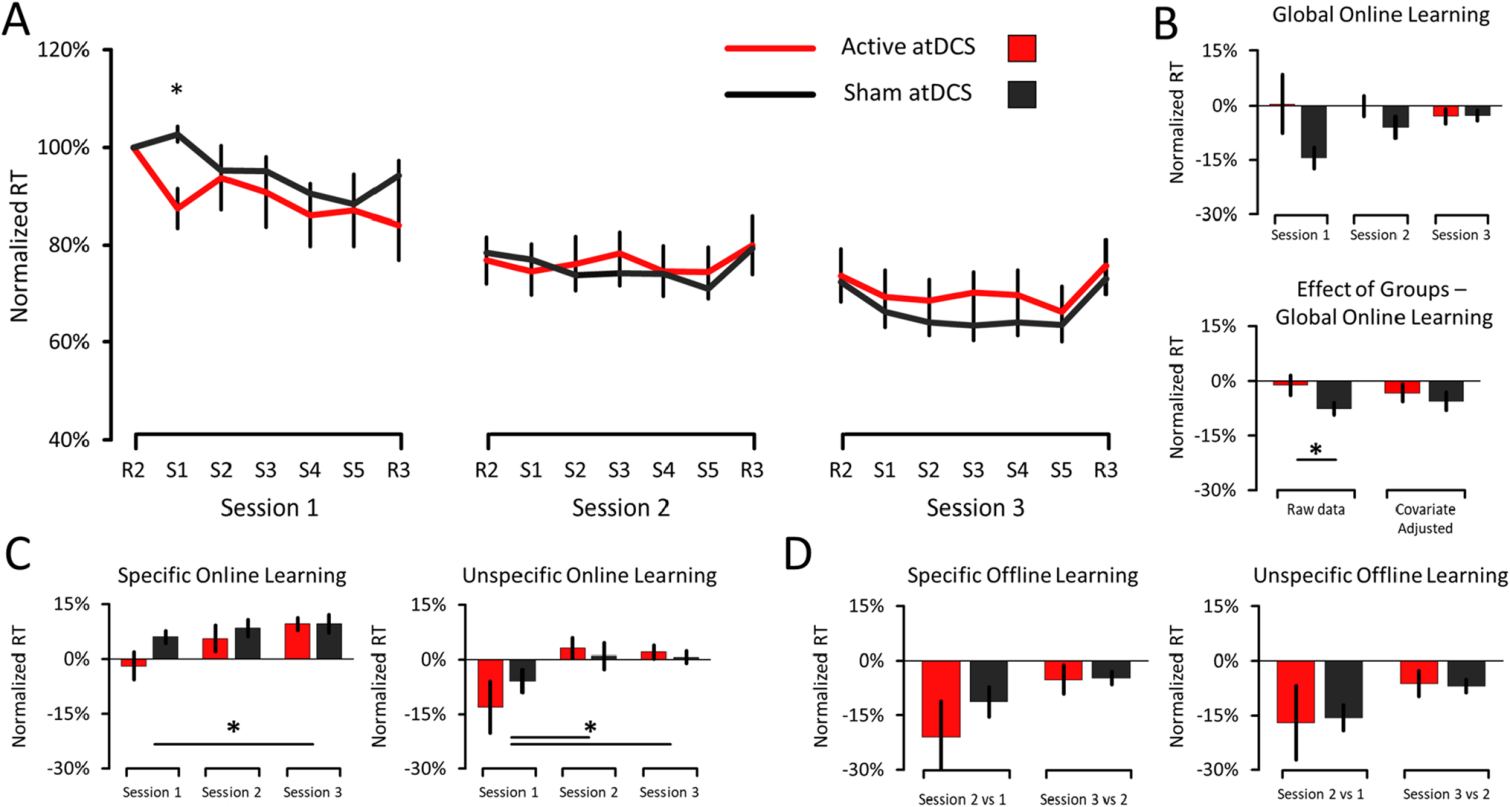
RT data of the SRTT. **a** Time-course of normalized RT across the three sessions. **b** *Upper Panel*: Global Online Learning data (S5–S1 of each session). *Lower Panel*: Effect of Groups in Global Online Learning data. Analyses of the raw data revealed an Effect of Groups (*p* = 0.052) where the Sham atDCS improved more than the Active atDCS group. However, when the significant difference observed at S1 (Session 1) is used as a covariate in the analyses, the effect of Group is no longer present (*p* = 0.552). **c** *Left Panel:* Specific Online Learning (R3–S5 of each session). *Right Panel:* Unspecific Online Learning (R3–R2 of each session). Main effect of Sessions were present in both Specific (*p* = 0.020) and Unspecific Online Learning (*p* = 0.001). **d** *Left Panel:* Specific Offline Learning data (S1–S5 of the preceding session). *Right Panel:* Unspecific Offline Learning data (R2–R3 of the preceding session). Error bars represent SEM. Asterisks (*) indicate significant differences (*p* ≤ 0.05). Note that the legend in **a** applies to every panel

The RT data are shown in Fig. 3. Concerning Global Online Learning, the results revealed an effect of Groups (F_(1,18)_ = 4.310, *p* = 0.052, ${n}_{p}^{2}$ = 0.193), but no effect of Sessions (F_(2,36)_ = 0.650, *p* = 0.484, ${n}_{p}^{2}$ = 0.035) and no interaction (F_(2,36)_ = 1.260, *p* = 0.295, ${n}_{p}^{2}$ = 0.066). The effect of Groups revealed that the Sham atDCS (− 7.72 ± 1.63%) improved their RTs more than the Active atDCS (− 1.16 ± 2.71%), suggesting that cerebellar atDCS impaired rather than improved learning during the SRTT. However, this result may be confounded by the significant difference presence between Groups at the S1 block of the first session (*t*_(18)_ = 3.464, *p* = 0.003, Cohen’s d = 1.549), where the Active atDCS (87.46 ± 4.09%) showed lower RT than the Sham atDCS group (102.70 ± 1.57%). When this confounding difference is used as a covariate, the results on the two-way ANOVA no longer indicated an effect of Groups (F_(1,17)_ = 0.368, *p* = 0.552, ${n}_{p}^{2}$ = 0.021). Specifically, the Sham atDCS (− 5.61% ± 2.45%) no longer improved their RTs more than the Active atDCS (− 3.27% ± 2.45%), suggesting that the difference at the S1 block was driving the Group effect. Globally, taken at face value, the results suggest that active atDCS impaired rather than improved SRTT learning. However, when taking into account the group difference present at S1 of the first session, results indicate that the two groups improved similarly. These results suggest that active atDCS did not conclusively improve RTs during Global SRTT learning as compared to sham atDCS.

### RT Data: Similar Online and Offline Learning Improvements Between Groups

Regarding Specific Online Learning, the results revealed a marginal effect of Groups (F_(1,18)_ = 3.130, *p* = 0.094, ${n}_{p}^{2}$ = 0.148), an effect of Sessions (F_(2,36)_ = 4.390, *p* = 0.020, ${n}_{p}^{2}$ = 0.196), but no interaction (F_(2,36)_ = 1.315, *p* = 0.281, ${n}_{p}^{2}$ = 0.068). The marginal effect of Groups shows that the Sham atDCS (7.92 ± 1.50%) tended to display higher RTs than the Active atDCS group (3.98 ± 1.65%), suggesting that the Active atDCS slowed their RTs less during the R3 random blocks than the Sham atDCS group. On the other hand, the effect of Sessions indicated that Specific learning improved from the first to the third session (*t*_(19)_ = 3.121, *p* = 0.018, Cohen’s dz = 0.698), but neither between the first and second sessions (W = 76, *p* = 0.441, Cohen’s dz = 0.368) nor between the second and third sessions (*t*_(19)_ = 1.065, *p* = 0.300, Cohen’s dz = 0.238). Globally, these results indicate that the Active atDCS tended to show less Specific learning than the Sham atDCS group, which suggests similar online learning improvements between the two groups.

Concerning Unspecific Online Learning, the results revealed no effect of Groups (F_(1,18)_ = 0.373, *p* = 0.549, ${n}_{p}^{2}$ = 0.020), an effect of Sessions (F_(2,36)_ = 7.969, *p* = 0.004, ${n}_{p}^{2}$ = 0.307), but no interaction (F_(2,36)_ = 1.781, *p* = 0.183, ${n}_{p}^{2}$ = 0.090). The effect of Sessions revealed that RTs improved from the first to both the second (*t*_(19)_ = 3.083, *p* = 0.009, Cohen’s dz = 0.689) and third sessions (*t*_(19)_ = 2.873, *p* = 0.015, Cohen’s dz = 0.643), but not from the second to the third sessions (W = 118, *p* = 0.648, Cohen’s dz = 0.064). This suggests that both Groups improved similarly from the first session regarding unspecific learning. Concerning both Specific and Unspecific Offline Learning, the results revealed no effect of Groups, Sessions, and interaction (all F < 2.514, all *p* > 0.130, all ${n}_{p}^{2}$ < 0.123). Globally, these results indicate that the Active atDCS group did not show greater offline learning (i.e., consolidation) than the Sham atDCS group, which suggests similar offline learning improvements between the two groups.

### Error Data: Active atDCS Tended to Perform Fewer Errors than Sham atDCS

**Fig. 4:**
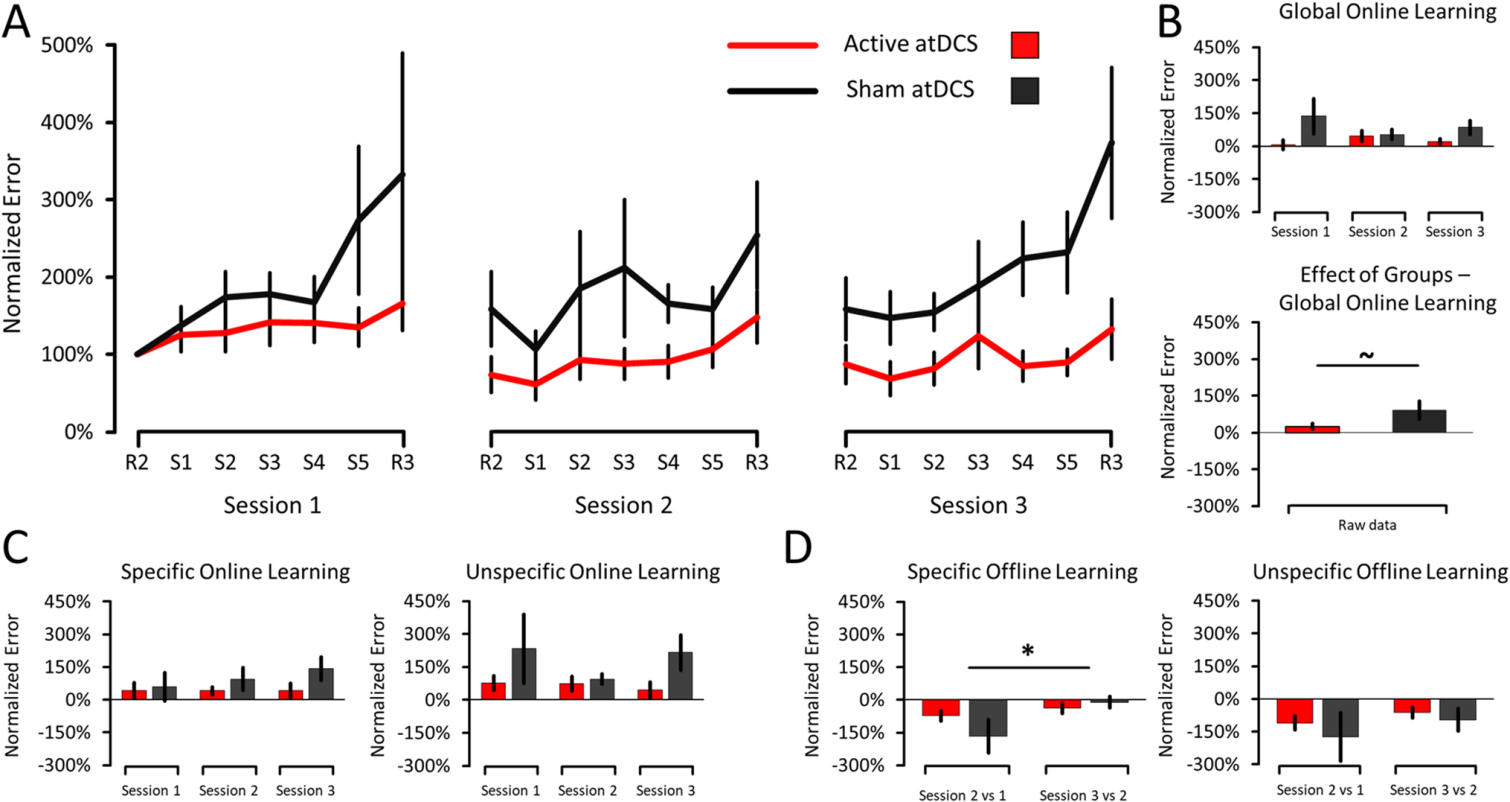
Error data of the SRTT. **a** Time-course of normalized Error across the three sessions. **b** *Upper Panel*: Global Online Learning data (S5–S1 of each session). *Lower Panel*: Effect of Groups in Global Online Learning data. The results revealed a marginal effect of Groups (*p* = 0.091) where the Active atDCS tended to commit fewer errors than the Sham atDCS group. **c** *Left Panel:* Specific Online Learning (R3–S5 of each session). *Right Panel:* Unspecific Online Learning (R3–R2 of each session). **d** *Left Panel:* Specific Offline Learning data (S1–S5 of the preceding session). The results revealed a significant effect of Sessions (*p* = 0.001). *Right Panel:* Unspecific Offline Learning data (R2–R3 of the preceding session). Error bars represent SEM. Asterisks (*) indicate significant differences (*p* ≤ 0.05). Tildes (~) indicate marginal differences (0.05 < *p* < 0.10 and when the resulting effect size is > large). Note that the legend in **a** applies to every panel

The Error data are shown in Fig. 4. Concerning Global Online Learning, the results revealed a marginal effect of Groups (F_(1,18)_ = 3.817, *p* = 0.091, ${n}_{p}^{2}$ = 0.150), no effect of Sessions (F_(2,36)_ = 0.218, *p* = 0.716, ${n}_{p}^{2}$ = 0.012), and no interaction (F_(2,36)_ = 1.166, *p* = 0.323, ${n}_{p}^{2}$ = 0.061). Breakdown of the marginal effect of Groups revealed that the Active atDCS (25.21 ± 11.15%) tended to perform fewer errors than the Sham atDCS group (91.06 ± 35.17%). Although not significant, the large effect size between the two groups suggests that cerebellar atDCS increased accuracy by decreasing the number of committed errors during Global SRTT learning.

### Error Data: Similar Online and Offline Learning Improvements Between Groups

Concerning Specific and Unspecific Online Learning, the results revealed no effect of Groups, Sessions, and interaction (all F < 1.845, all *p* > 0.191, all ${n}_{p}^{2}$ < 0.093), indicating that both Groups committed a similar amount of errors across sessions during Specific and Unspecific SRTT learning. Globally, this suggests similar online learning improvements between the two groups.

Concerning Specific Offline Learning, the results revealed no effect of Groups (F_(1,18)_ = 0.587, *p* = 0.454, ${n}_{p}^{2}$ = 0.032), an effect of Sessions (F_(1,18)_ = 4.228, *p* = 0.055, ${n}_{p}^{2}$ = 0.190), and no interaction (F_(1,18)_ = 1.665, *p* = 0.213, ${n}_{p}^{2}$ = 0.085). The effect of Sessions revealed that the number of errors decreased from the first contrast (Session 2 vs Session 1; − 120.26 ± 41.84%) to the second one (Session 3 vs Session 2; − 24.73 ± 17.48%). This indicates that both groups showed greater specific offline improvements between the first and second sessions than between the second and third sessions. Concerning Unspecific Offline Learning (R2–R3 of the preceding session), the results revealed no effect of Groups, Sessions, and interaction (all F_(1,18)_ < 1.309, all *p* > 0.268, all ${n}_{p}^{2}$ < 0.068). Globally, this suggests similar offline learning improvements between the two groups.

### Similar Performance Levels Between Groups During the FNT

Concerning the FNT Time data, the results revealed a Sessions X Times (Pre, Post) interaction (F_(2,36)_ = 8.520, *p* = 0.003, ${n}_{p}^{2}$ = 0.321), but no effect or interaction involving the Groups factor (all F < 1.728, all *p* > 0.205, all ${n}_{p}^{2}$ < 0.088), which indicates that application of cerebellar active atDCS did not facilitate performance during the FNT. Breakdown of the interaction revealed that participants improved from Pre to Post in Session 1 (*t*_(20)_ = 4.142, *p* = 0.002, Cohen’s dz = 0.926), but neither in Session 2 (*t*_(19)_ = 0.019, *p* = 0.985, Cohen’s dz = 0.004) nor Session 3 (*t*_(19)_ = 1.213, *p* = 0.360, Cohen’s dz = 0.271). Concerning the FNT Number data, the results revealed no significant interaction or main effect (all F < 2.351, all *p* > 0.110, all ${n}_{p}^{2}$ < 0.115). Overall, these results show that participants improved the Time needed to perform the FNT selectively in their first Session and that both Groups did not differ, suggesting that cerebellar atDCS application did not influence FNT performance.

## Discussion

The objective of this study was to assess the effects of cerebellar atDCS on motor sequence learning and coordination in children with DCD. First, initial results revealed that the Sham atDCS group improved their Global RT more than the Active atDCS group, suggesting that active cerebellar atDCS impaired sequence learning. However, the inclusion of the group difference at S1 (Session 1; Fig. 3) as a covariate mitigated this result by revealing that the Active and Sham atDCS groups no longer differed. Together, these results suggest that active cerebellar atDCS did not conclusively enhance RT improvements during sequence learning. Second, the results revealed that the Active atDCS group tended to commit fewer errors than the Sham atDCS group, suggesting that cerebellar atDCS improves accuracy during sequence learning. Third, the results revealed no difference between the Active and Sham atDCS groups in all measurements of Offline Learning and the FNT, suggesting that cerebellar atDCS did neither improve motor memory consolidation nor upper limb motor coordination, respectively. One discussed possibility is that cerebellar atDCS primarily stimulated the cerebellum’s posterior lobe, which is involved in the regulation of higher cognitive functions rather than motor learning and coordination (Schmahmann, 2019). This could explain why cerebellar atDCS selectively improved accuracy but neither learning speed, motor memory consolidation, nor upper limb coordination.

### No Conclusive Effects of Cerebellar atDCS on Sequence Learning Speed

One surprising initial result was that the Sham atDCS group improved its Global RT more than the Active atDCS groups, suggesting that cerebellar atDCS was detrimental to sequence learning. However, given that Global Online Learning is calculated as the difference between the S1 and S5 blocks of each session, this result was likely confounded by the large difference observed between groups at S1 of the first session (Fig. 3A). The inclusion of this difference as a covariate mitigated this result by showing that the Sham and Active atDCS groups no longer differed. As a result, contrary to one of this work’s hypotheses, these results do not conclusively indicate that cerebellar atDCS enhanced overall sequence learning speed.

These results are arduous to put in relation with previous studies, as mixed result patterns have been reported on the influence that cerebellar atDCS should have on sequence learning. Namely, previous work has shown that cerebellar atDCS impairs (Jongkees et al., 2019), does not affect (Ballard et al., 2019), or enhances (Shimizu et al., 2017) motor sequence learning as compared to Sham conditions. Interestingly, a recent meta-analysis of functional neuroimaging data revealed that sequence learning relies on the basal ganglia and that the cerebellum does not directly contribute to sequence learning itself (Janacsek et al., 2020), which suggests that the cerebellum may not be the key target to enhance motor sequence learning in healthy humans. However, since this structure–function evidence is correlational, that the causal results are mixed in healthy humans, and that this is the first study evaluating the effects of cerebellar atDCS in DCD children, the contribution of the cerebellum to sequence learning in DCD children remains to be ascertained by future confirmatory studies.

### Cerebellar atDCS Tended to Improve Accuracy

The results revealed that the Active atDCS group tended to perform fewer errors than the Sham atDCS group during Global Online Learning, suggesting that cerebellar atDCS tended to improve accuracy during sequence learning. This result dovetails previous results (Cantarero et al., 2015; Ehsani et al., 2016) but also opposes others (Ballard et al., 2019) reported in healthy humans. Specifically, on the one hand, Cantarero et al. (2015) found that cerebellar atDCS enhanced motor learning and retention of a force pinch task through an effect of accuracy. On the other hand, Ballard et al. (2019) found that cerebellar atDCS impaired accuracy while cerebellar cathodal tDCS—a putatively inhibitory NIBS technique—facilitated accuracy during motor sequence learning, suggesting that inhibiting the cerebellum is beneficial to sequence learning. As a result, it remains uncertain if cerebellar atDCS can effectively improve accuracy during motor learning in healthy humans. Although awaiting further confirmation, the present results nonetheless indicate that cerebellar atDCS can enhance accuracy in DCD children during sequence learning. This suggests that cerebellar atDCS can be used as an adjuvant to motor training therapies to facilitate DCD rehabilitation.

### Cerebellar atDCS Did Not Enhance Offline Learning

The results revealed that the Active atDCS group did not show greater Offline Learning—either Specific or Unspecific for both RTs and Errors—than the Sham atDCS group, suggesting that cerebellar atDCS did not enhance motor memory consolidation. Here as well, previous work indicated mixed result patterns by showing that cerebellar atDCS enhances (Cantarero et al., 2015; Jalali et al., 2018; Shimizu et al., 2017; Wessel et al., 2016), does not influence (Galea et al., 2011) or is detrimental to offline learning (Jongkees et al., 2019), which makes it arduous to infer on the expected influences of atDCS on motor memory consolidation in healthy humans. Here, the present results suggest that the effects of atDCS on performance were temporally circumscribed to its time of application, with little to no lingering performance aftereffects once atDCS was switched off.

However, this possibility is difficult to reconcile with evidence showing atDCS-induced structural changes in gray and white matter (Hirtz et al., 2018), which suggests that the effects of atDCS can outlast its time of application. Moreover, when learning is considered from a synaptic perspective (Baltaci et al., 2019), interventions that are presumed to facilitate synaptic plasticity should translate, at least partly, to enduring synaptic changes. Functionally, this implies that the online performance improvements induced by cerebellar atDCS should also yield qualitatively similar offline performance improvements. However, given that results in healthy adults are mixed and that the present results do not suggest offline effects of cerebellar atDCS on performance in DCD children, alternative interpretations are warranted.

Another possibility is that the cerebellar neural structures targeted by atDCS mainly regulate higher cognitive functions—such as attention and working memory—without directly contributing to motor coordination and learning (for a recent review, see Schmahmann (2019). Namely, while the cerebellum’s anterior lobe is responsible for motor coordination and learning, its posterior lobe has widely distributed ramifications to associative areas, including the prefrontal cortex, and is actively involved in the regulation of higher cognitive functions (Buckner, 2013; Schmahmann, 2019). In light of modeling studies showing that the strongest cerebellar tDCS electric fields reach the posterior lobe due to its proximity to the scalp (Parazzini et al., 2014), here, one possibility is that cerebellar atDCS predominantly stimulated the cerebellum’s posterior lobe and thus preferentially enhanced cognitive functions that support—without directly mediating—sequence learning. This possibility resonates with a very recent meta-analysis showing that the cerebellum supports, but does not directly contribute to sequence learning (Janacsek et al., 2020). This could explain why the online effects of atDCS were selective to accuracy and did not translate to similar offline improvements. Whether cerebellar atDCS contributes to motor coordination and learning through an improvement of higher cognitive functions should be examined by future studies.

### No Meaningful Effects of Cerebellar atDCS on Upper Limb Coordination

The present results revealed that the Active atDCS group did not differ from the Sham atDCS group in terms of performance at the FNT, suggesting that cerebellar atDCS did not improve upper limb coordination in DCD children. Considering that this work is the first to assess the effects of cerebellar atDCS in DCD children, it is difficult to determine if this result is attributable to the disorder itself, to characteristics inherent to brain maturation, or the stimulation protocol used. Moreover, the effects of cerebellar atDCS on motor coordination in ataxic patients—patients suffering from impaired motor coordination similar to DCD children—remains controversial (Benussi et al., 2020; Grimaldi & Manto, 2013). Interestingly, recent studies indicate that repeated application of cerebellar atDCS may be key to optimize the outcomes of such interventions (Benussi et al., 2017; Pilloni et al., 2019). Namely, Benussi et al. (2017) found that 2 weeks of cerebellar atDCS application, as compared to 3 days, improved motor coordination of ataxic patients, including their FNT scores. Here, one possibility is that 3 days of cerebellar atDCS were insufficient to yield meaningful improvements of upper limb coordination in DCD children. Further clarifications will be required to determine if repeated applications of cerebellar atDCS can optimize functional upper limb coordination gains in DCD children.

### Study Limitations

First, factors such as the higher prevalence of co-morbid neurodevelopmental disorders and the presence of psychoactive medication in our sample may have influenced responsiveness to cerebellar atDCS. However, given our small sample size, it was unfeasible to stratify children according to such factors. Second, motor learning acquisition in children with DCD and the effects of cerebellar atDCS on motor learning each depend on many elements such as motivation and fatigue (Wulf et al., 2010; Zwicker et al., 2010). Children with DCD tend to be more anxious than other children (Pratt & Hill, 2011) and commonly report fatigue during the performance of motor tasks (Zwicker et al., 2010). These factors may have impacted motivation and perseverance during SRTT, which is a potential source of confound. Thirdly, the brain regions involved in DCD have been reported to vary between individuals (Farmer et al., 2017). Targeting the same brain region with atDCS without any regard for the heterogeneity in the neuroanatomical bases of DCD could account for the present results. One possibility is that atDCS would yield greater effects if it is targeting individually pre-identified brain areas (Benussi et al., 2020). Finally, the sensitivity of the FNT to assess the effects of atDCS on upper limb motor coordination remains unknown, which may explain the present absence of group differences. Further studies are needed to explore how can putative changes of cerebellar excitability result in motor improvements and clarify their effects on distal brain regions, such as the primary motor cortex (see Schlerf et al., 2012). Furthermore, the effect of different electrode montage and neuromodulation techniques (i.e. transcranial magnetic stimulation) in improving motor function of children with DCD should be investigated along with the potential gains of using neuromodulation as an adjuvant to intensive motor training therapies.

## Conclusion

The results of this study show that cerebellar atDCS did not improve online sequence learning speed, but tended to improve online accuracy in children with DCD. Whether or not these online improvements can also lead to enduring offline improvements or functional gains in upper limb coordination remains to be determined. Additional research with larger sample sizes is needed to establish if atDCS can be a valuable complementary therapeutic tool for the rehabilitation of children with DCD.
